# Workmen's Subscriptions and Representation

**Published:** 1912-11-30

**Authors:** Tom Morgan

**Affiliations:** Secretary. Pontypool and District Hospital


*To the Editor of* THE HOSPITAL.

### Workmen's Subscriptions and Representation.

*To the Editor of* THE HOSPITAL.

SIR,—In the report of the Royal Victoria Infirmary, Newcastle, Sir Henry Burdett, referring to the rule of electing governors—*i.e.*, that for every £10 contributed by the workmen they are entitled to nominate a governor to attend the meetings of the infirmary. He believes it is the only example in the country where the working classes, by their contributions, have the right to nominate nearly 1,900 governors in any year. I may state it has been the rule at our annual courts from its inception.

The workmen contribute one penny each per week; for every £10 contributed they are entitled to appoint one of their number to attend the court of governors for the following year. In consequence of this rule all the workmen within the area covered by the hospital are willing subscribers. Their employers supplement their employees' contributions by 25 per cent. This rule of representation is applicable to all other sections of contributors. It has been most satisfactory, both administratively and financially. All contributing interests are represented on the executive board; we are not hampered in our work for the want of funds, although for eight weeks in the early part of the year our chief source of income was stopped owing to the coal strike, the balance with the treasurer will allow of some surplus being invested as a reserve for any future requirements.—Yours faithfully,

TOM MORGAN, Secretary.

Pontypool and District Hospital,
November 25.

[The Hospital contains 36 beds.—ED. THE HOSPITAL.]

